# Effects of movement congruence on motor resonance in early Parkinson’s disease

**DOI:** 10.1038/s41598-023-42112-2

**Published:** 2023-09-09

**Authors:** Eleonora Gentile, Antonio Brunetti, Katia Ricci, Eleonora Vecchio, Carlo Santoro, Elena Sibilano, Vitoantonio Bevilacqua, Giovanni Iliceto, Laila Craighero, Marina de Tommaso

**Affiliations:** 1 Neurophysiopathology Unit, Polyclinic General Hospital, University of Bari “Aldo Moro”, 70136 Bari, Italy; 2 Department of Electrical and Information Engineering, Polytechnic University of Bari, 70125 Bari, Italy; 3 Department of Neuroscience and Rehabilitation, University of Ferrara, 44121 Ferrara, Italy

**Keywords:** Parkinson's disease, Neuroscience, Neurological disorders

## Abstract

The observation of action seems to involve the generation of the internal representation of that same action in the observer, a process named motor resonance (MR). The objective of this study was to verify whether an experimental paradigm of action observation in a laboratory context could elicit cortical motor activation in 21 early Parkinson’s disease (PD) patients compared to 22 controls. Participants were instructed to simply observe (observation-only session) or to respond (Time-to-contact detection session) at the instant the agent performed a grasping action toward a graspable or ungraspable object. We used functional near-infrared spectroscopy with 20 channels on the motor and premotor brain areas and event-related desynchronization of alpha-mu rhythm. In both groups, response times were more accurate in graspable than ungraspable object trials, suggesting that motor resonance is present in PD patients. In the Time-to-contact detection session, the oxyhemoglobin levels and alpha-mu desynchronization prevailed in the graspable object trials rather than in the ungraspable ones. This study demonstrates the preservation of MR mechanisms in early PD patients. The action observation finalized to a consequent movement can activate cortical networks in patients with early PD, suggesting early rehabilitation interventions taking into account specific observation paradigms preceding motor production.

## Introduction

The role of the motor cortex has long been known in cognitive processes such as, for example, motor planning, motor imagination, perception of action, and motor learning. The observation of action seems to involve the generation of the internal representation of that same action in the observer, a process named Motor Resonance (MR)^1,2^. Importantly, action observation determines the activation of different networks located in the visual, motor, and perceptive areas^3^.

Understanding the neurophysiological mechanisms of action observation effects on the brain of neurological patients has been a hot topic in the last few years. Specifically, the progressive aging of the population poses new challenges to rehabilitation medicine, in particular for those neurodegenerative and disabling diseases such as Parkinson's disease (PD)^4^.

PD represents a neurodegenerative disease noted by a complex impairment of motor behavior. Previous neuroimaging evidence demonstrated the involvement of basal ganglia in action observation tasks^5^, suggesting, therefore, an influence of the motor dysfunction in PD on the internal representation of the action. People with Parkinson's disease appear to have a slowdown in the form of motor learning, probably due to impairment in executive functions^6^. Recent neuroimaging studies reported a defect in brain areas during observation of gait in Parkinson’s disease patients^3^. The basal dysfunction of PD may play a role in the functioning of motor resonance mechanisms. Motor cognition appears to represent a promising field of study for the design of rehabilitation interventions for patients with PD, including those based on action observation^4^.

The main objective of this study was to verify whether an experimental paradigm of action observation in a laboratory context could elicit cortical motor activation in PD patients. For this purpose, PD patients and sex- and age-matched controls were enrolled in the present study. To the best of our knowledge, in this work, such a paradigm is applied for the first time to PD subjects in order to investigate how the congruence of a movement could affects mirror mechanisms. We used a functional analysis based on metabolic and EEG graphic changes induced by a robust action prediction paradigm^7^ already proved to be effective in providing indirect evidence for the presence of motor resonance^7–11^. Participants observed videos of grasping actions directed towards a graspable or an ungraspable object. They were instructed to respond the instant the agent touched the object (Time-to-contact detection session); in fact, an indirect indicator of motor resonance is the occurrence of more precise responses for the graspable object trials. In a different experimental session, instead, participants were instructed to watch and pay attention to the videos (Observation-only session). During each experimental session, the participants’ cerebral hemodynamic activity was recorded using a functional Near-Infrared Spectroscopy (fNIRS) with 20 channels located on the motor and premotor brain areas. Furthermore, an EEG analysis, focused on event-related desynchronization of alpha rhythm (alpha-mu rhythm), was considered to verify the presence of a sensorimotor network involvement.

We predicted more precise responses (i.e., faster responses) to graspable object trials in control participants, supporting prior findings^7–11^. A comparable pattern in the responses of PD patients would indicate the presence of normal motor resonance. We also expected possible differences between PD patients and controls in the metabolic tone of the motor network and mu rhythm suppression during movement observation and in preparation for motor behaviour.

## Results

### Behavioural data

**Figure 1:**
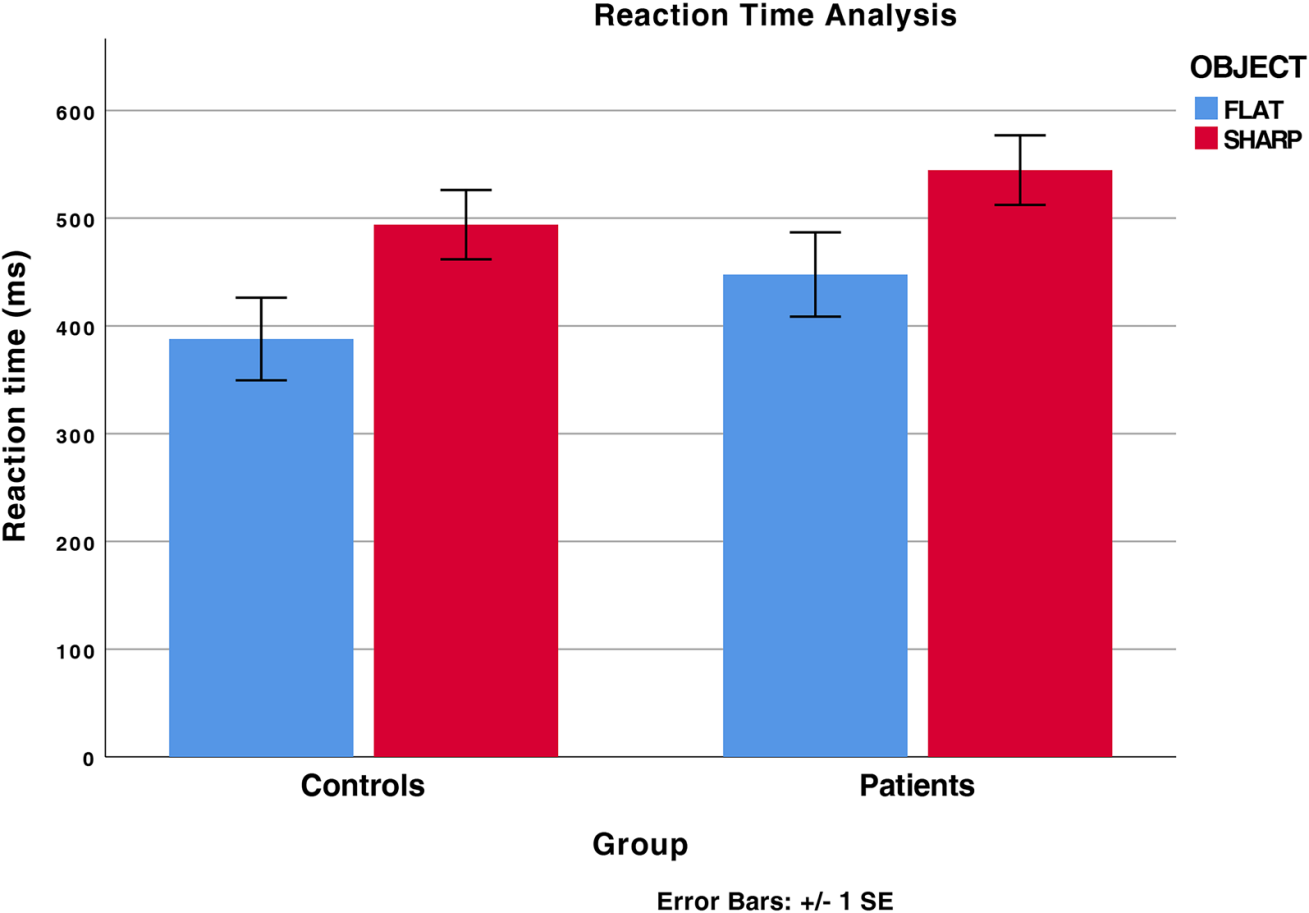
Detection time results. Time lag between the instant at which the agent touches the object and participant’s response time. For both groups (P, Parkinson patients; C, Controls), data for flat object trials and sharp-tip object trials are shown. Thin lines above histograms indicate standard error of the mean.

The results of the two-way ANOVA performed on the time lag showed that the object main effect was significant (F = 8.114, *p* < 0.006, η_p_^2^ = 0.101). The time lag in flat object trials (controls mean = 387.87 ms, SEM = 38.36 ms; patients mean = 447.73 ms, SEM = 39.11 ms) was shorter than in sharp tip object trials (controls mean = 439.97 ms, SEM = 32.15; patients mean = 544.59 ms, SEM = 32.30 ms). The factor group was not significant (F = 2.404, *p* = 0.125), as well as the 2-way interaction group *x* object (F = 0.17, *p* = 0.897). The detection time results were reported in Fig. 1. These results suggest that response times were modulated by the object’s graspability in both PD patients and controls. Therefore, behavioural data suggest that both groups exhibit motor resonance.

### fNIRS data

#### Resting-state: comparison between time preceding time-to-contact detection session and observation-only session

In the resting-state condition, no differences in oxyhemoglobin levels were detectable between groups in relation to the time preceding the observation-only session and the time-to-contact detection session.

#### Time-to-contact detection session: comparison between flat object trials versus sharp-tip object trials

**Figure 2:**
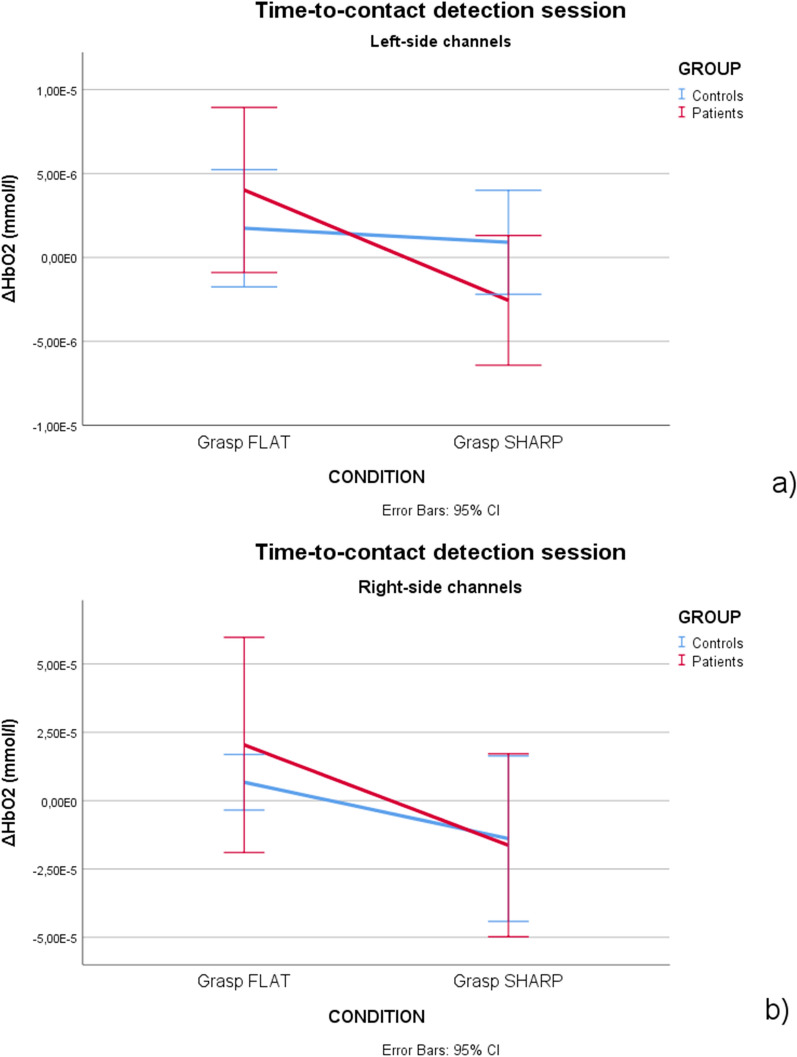
Mean and 95% CI of oxyhemoglobin levels. In (**a**) the left channels in controls (blue color) and PD patients (red color). During the Time-to-contact detection task, oxyhemoglobin increased in a more evident way in PD patients group during the grasp flat object trial. On the right channels (**b**), the oxyhemoglobin increase during the grasp flat object trial was evident in both groups.

**Figure 3:**
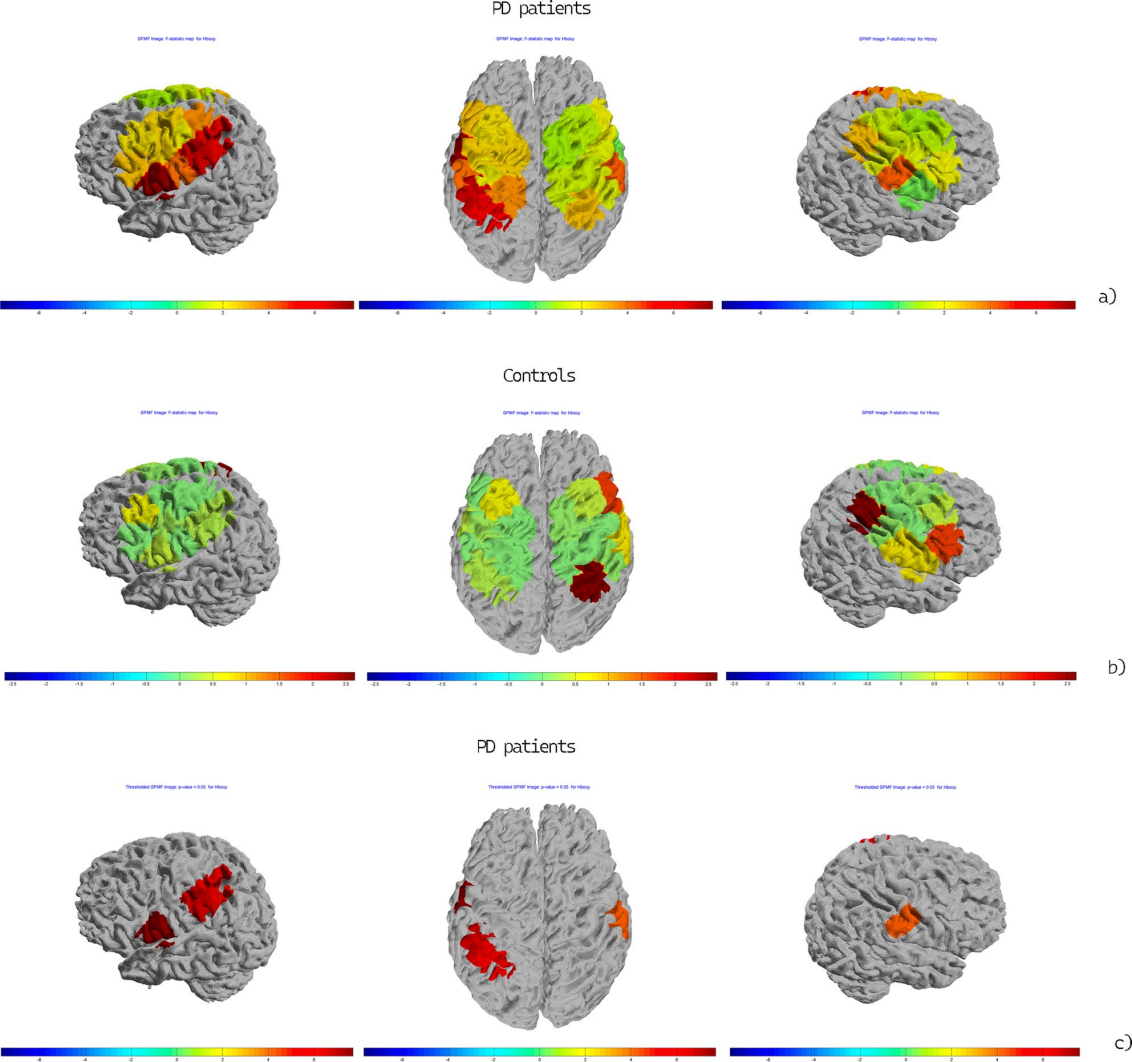
Topographic maps of the F-statistic in the comparison between conditions (Grasping Flat vs Grasping Sharp-tip object) in the PD patients (**a**) and controls (**b**) in Time-to-contact detection session. In (**c**) a particular of the F-statistic for the significant channels (Ch 7, Ch 8, Ch 10, *p* < 0.05) in the comparison between conditions (Grasping Flat vs Grasping Sharp-tip object) in the PD patient group for the time-to-contact session is reported. The color map represents the value of the F-statistic.

In the time-to-contact detection session, the Object main effect was significant both in the left (F = 4.03,* p* = 0.048, η_p_^2^ = 0.044) and right (F = 4.12, *p* = 0.045, η_p_^2^ = 0.045) channels, revealing an increase of oxyhemoglobin levels during flat object trials compared to sharp-tip object trials. PD patients showed a tendency through a more evident increase in oxyhemoglobin levels for the vision of the flat object, but the difference did not reach statistical significance (Fig. 2). The statistical map reporting significant changes in single channels confirmed increased activation on the left and right hemispheres in patients, and on the right hemisphere in controls during the vision of flat object (Fig. 3).

#### Observation-only session: comparison between flat object trials versus sharp-tip object trials

**Figure 4:**
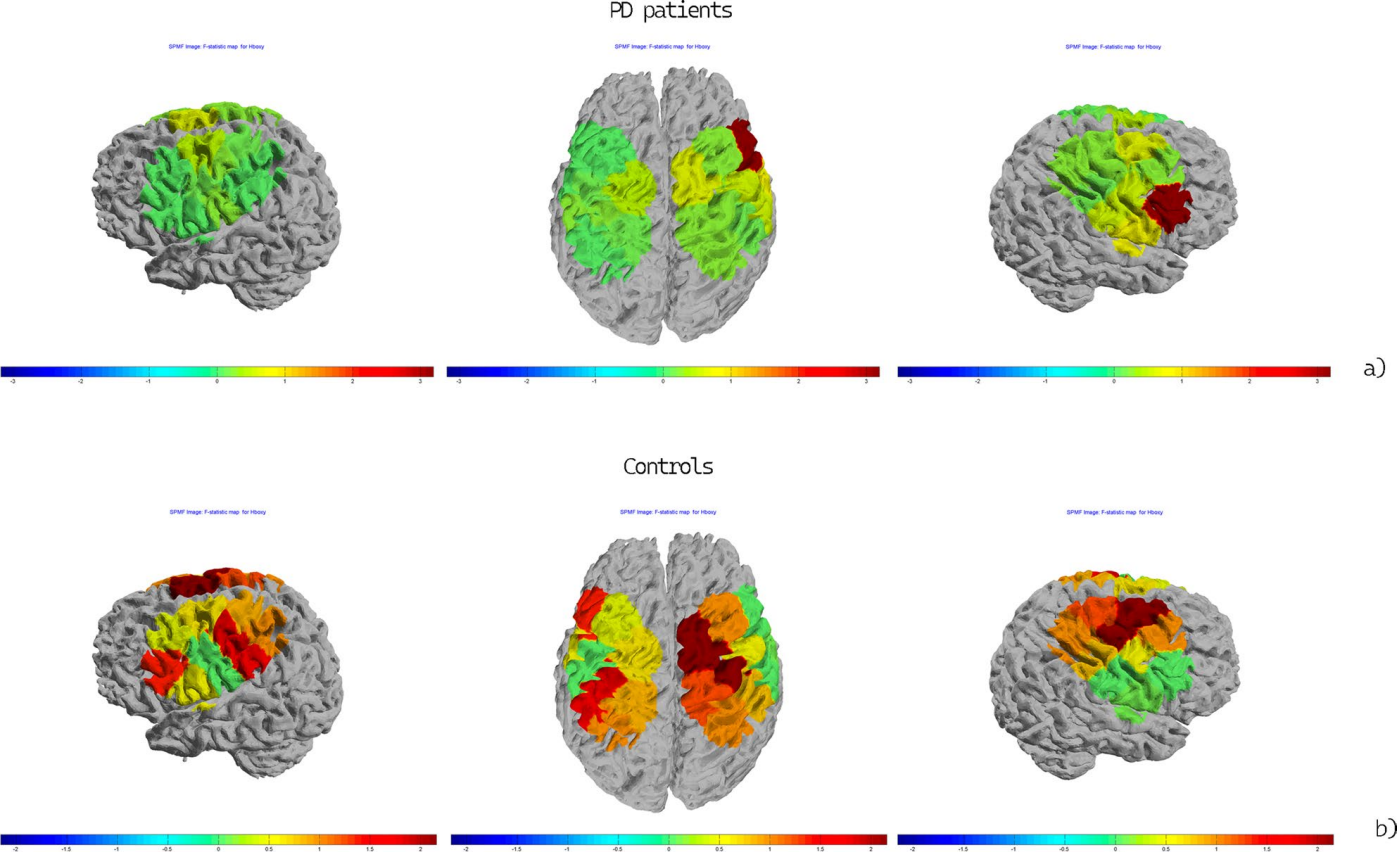
Topographic maps of the F-statistic in the comparison between conditions (Grasping Flat vs Grasping Sharp-tip object) in the PD patient group (**a**) and controls (**b**) for the observation-only session. The color map represents the value of the F-statistic.

In the observation-only session, no main effect or interaction in oxyhemoglobin levels was significant for either the left or right channels. The single channels topographic analysis showed increased activation in a small right hemisphere region, corresponding to 1 channel, in PD patients, during flat object trials compared to sharp-tip object trials (Fig. 4a). In controls, the same phenomenon was expressed in both hemispheres (Fig. 4b).

### EEG results

#### ALPHA mu: comparison between time preceding time-to-contact detection session and observation-only session

For the resting-state, we considered the 5 s preceding the time-to-contact detection session and the observation-only session.

**Figure 5:**
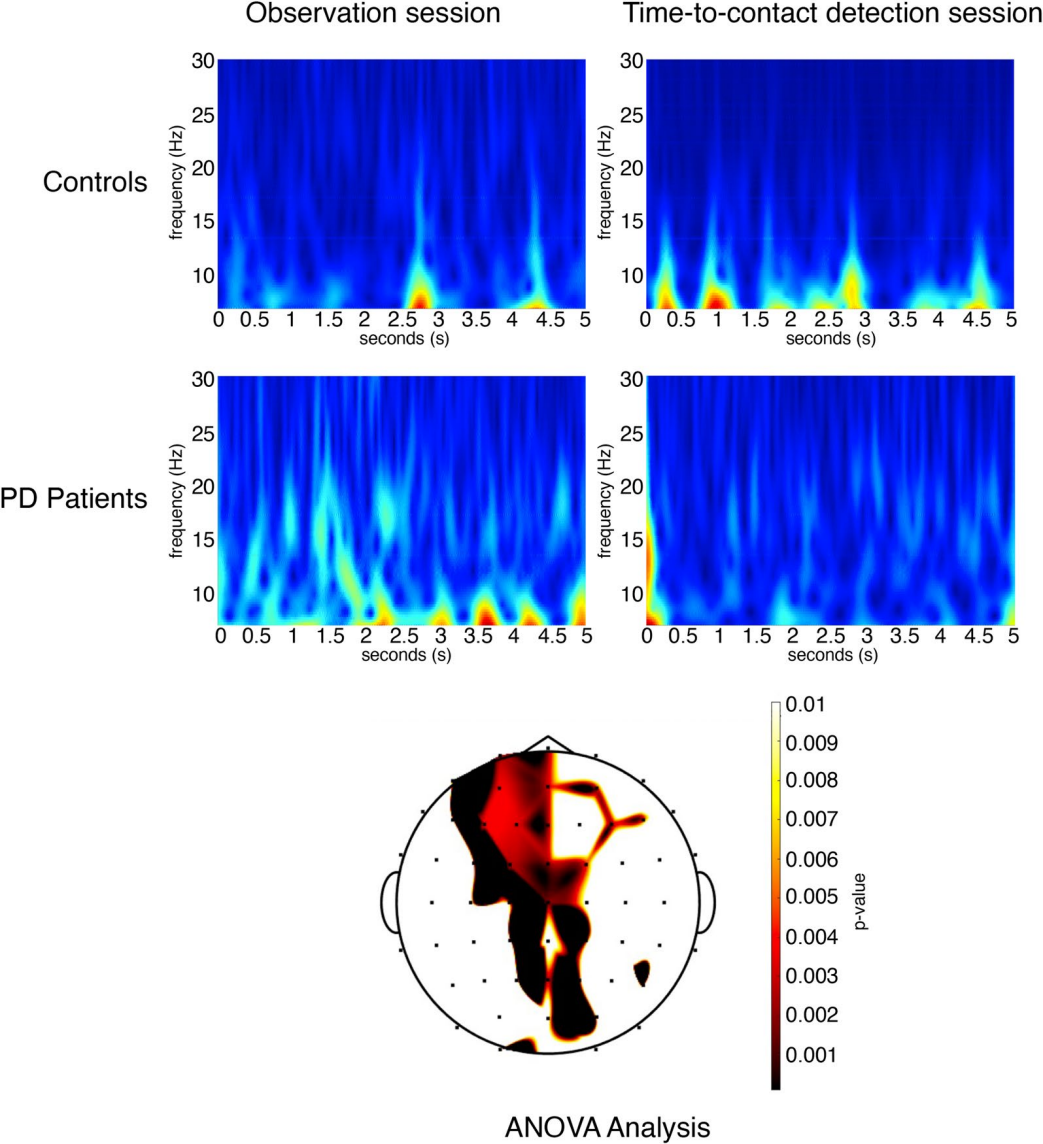
Resting-state preceding the observation and Time-to-contact detection session. (Up) The Grand Average of Continuous Wavelet Transform of alpha-mu recorded on the C3 derivation in the 5 s of resting state preceding the observation session and Time-to-contact detection session is reported for controls and PD patients groups. In PD patients, desynchronization of EEG rhythm is evident in the 8–13 Hz range in the time preceding the Time-to-contact detection session, in controls desynchronization prevailed in the low alpha range before the observation-only session. (Bottom) The statistical map reports the *p*-values obtained with ANOVA analysis for the interaction group x session. It shows that alpha desynchronization was more evident in PD patients on the fronto-central electrodes for the effect of the session.

*Controls*. In controls, we observed that in the lower frequencies range, the alpha rhythm was more desynchronized in the time preceding the observation-only session (Fig. 5). However, this did not reach the statistical significance.

*PD patients.* In PD patients, the alpha mu desynchronized in the time preceding the time-to-contact detection session (Fig. 5). The t-test for paired data showed a significant desynchronization in the 13–18 Hz range over the central regions.

*Comparison between groups*. Alpha mu desynchronization was more evident in PD patients over the left fronto-central regions in the seconds preceding the time-to-contact detection session, as indicated by the results of the ANOVA test considering the session and groups as factors (Fig. 5).

#### Time-to-contact detection session: comparison between flat object trials vs sharp-tip object trials

**Figure 6:**
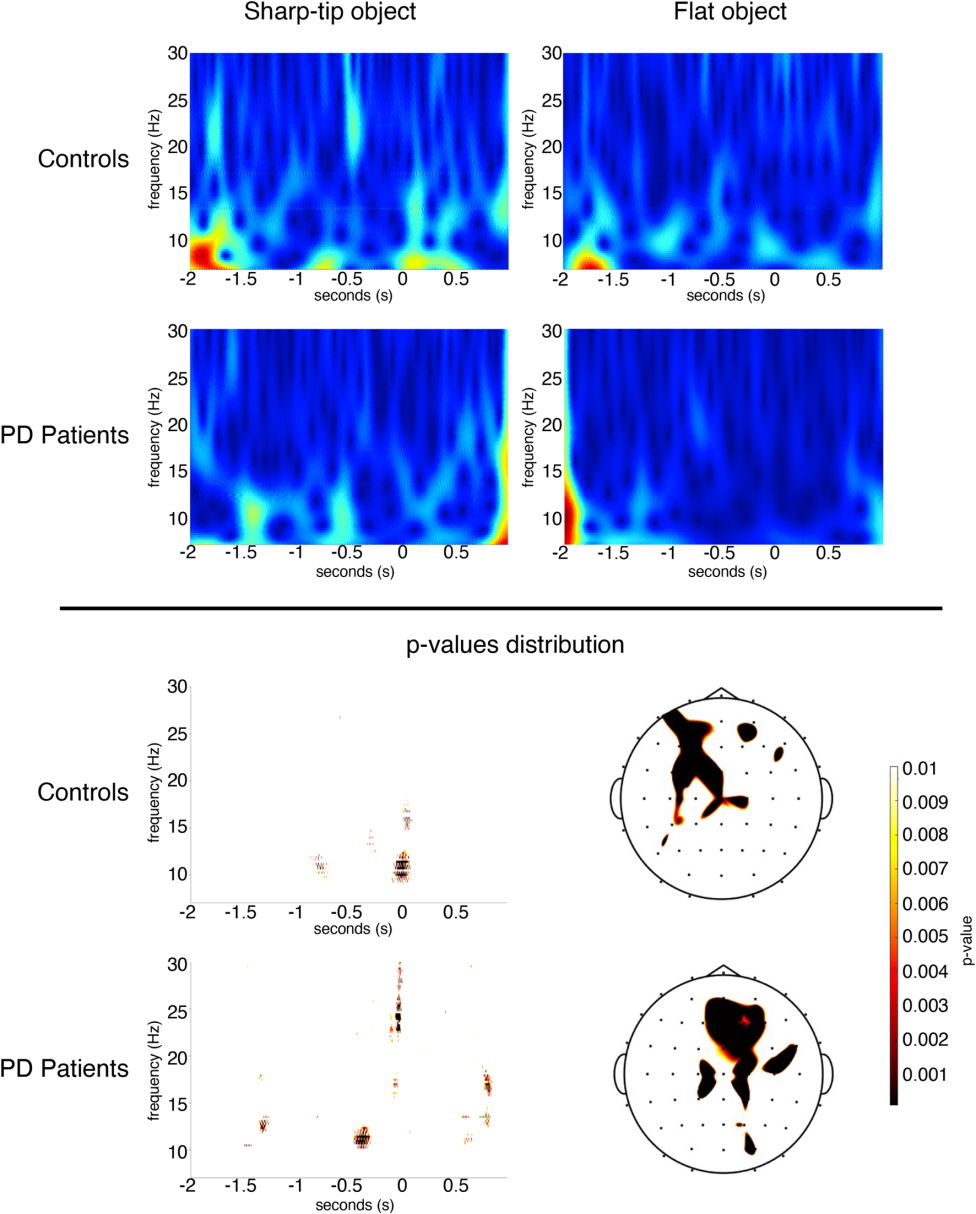
Time-to-contact detection session: comparison between flat vs sharp-tip object. (Up) The Grand Average of time–frequency analysis of alpha-mu recorded on the C3 derivation in the 2 s preceding and 1 s following the flat and sharp-tip object grasping are reported for controls and PD patients. (Bottom) For each group, the *p*-values obtained with paired t-test between flat vs sharp tip object are reported on the C3 channel, and on the statistical map. Before the flat object trials, we observed that alpha-mu desynchronization prevailed in the 8–9.5 Hz range in the 2 s time in controls, and in the 1 s time in the 11–13 Hz range in PD patients.

*Controls*. Desynchronization of the mu rhythm in the alpha range was present in the second preceding the grasp of the objects. Two seconds before the flat object grasp, the alpha rhythm desynchronization prevailed in the 8–12 Hz range, on the left fronto-central electrodes, compared to sharp-tip object (Fig. 6).

*PD patients.* In PD patients, the desynchronization of alpha mu was also present in the second preceding and following both objects grasping. We also observed desynchronization in the range 11–13 Hz in the same time preceding the hand grasping the flat object. The comparison between flat object trials vs sharp-tip object trials between groups was not significant (Fig. 6).

#### Observation-only session: comparison between flat object trials vs sharp-tip object trials

**Figure 7:**
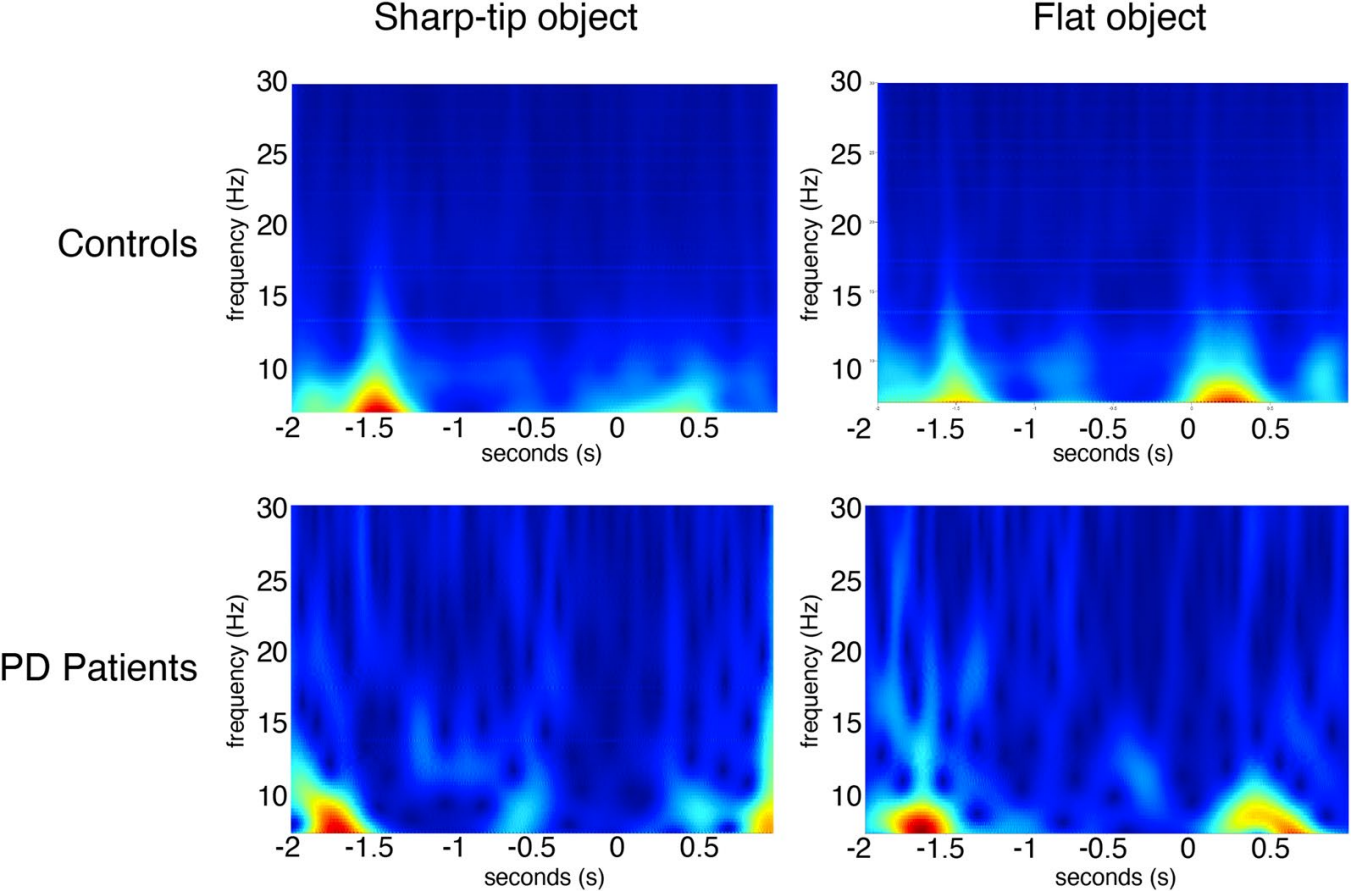
Observation-only session: comparison between flat vs sharp-tip object. The Grand Average of time–frequency analysis of alpha-mu recorded on the C3 derivation in the 3 s preceding and 1 s following the flat and sharp tip object grasping are reported in controls and PD patients.

*Controls*. The mu rhythm, especially in alpha range, showed a tendency to a desynchronization in the time preceding the grasp of both objects. In the time following the incongruent movement, the alpha rhythm appeared more desynchronized, though no significant change was detected with the t-test. (Fig. 7).

*PD patients.* Patients showed a similar mu rhythm desynchronization, especially in the alpha range, in approaching the objects grasping, though the two objects did not induce different mu rhythm behaviour.

The comparison across groups and conditions was not significant.

## Discussion

The present research evaluated the motor resonance mechanisms in PD patients compared to controls. Behavioural results revealed that response times in PD patients did not differ from those of control participants. In both groups, response times were more accurate (i.e., shorter time lag between the instant at which the agent’s touched the object and the participant’s key pressing) during flat object trials than during sharp tip object trials. This effect replicates previous results, and it is considered proof of the fact that during the observation of the action individuals automatically activate the related sensorimotor representation built on their own experience. When observing actions that the individual would never perform, the motor system is not involved. The lack of motor simulation prevents the same level of accuracy in time-to-contact detection that occurs when observing an action that we would have no problem performing ^7–11^. Therefore, present results suggest that motor resonance is present in PD patients. We did not find relevant differences in basal motor networks metabolism in PD patients as compared to controls, but in the time-to-contact detection session, we observed that the observation of the suitable grasping action (i.e., Flat object videos), preceding the detection response, favored cortical activation. In the resting state, the alpha mu desynchronization was more evident in PD patients than in controls. The video of the flat object grasping, preceding the behavioural response, caused alpha mu desynchronization in patients and controls, with respect to the sharp tip object. In the observation session, we observed that in control subjects the alpha mu rhythm was desynchronized with the appearance of sharp-tip object grasping, probably expressing a cortical activation in response to the imminent other’s wrong movement.

According to behavioral, metabolic, and EEG data, results indicate the preservation of motor resonance mechanisms in our early PD patients. However, some differences exist between PD patients and healthy subjects. In the following, the results will be discussed in detail.

### Resting-state

 Metabolic activity of the cortical motor network, as reported by the basal oxyhemoglobin levels, was similar between PD patients and controls. Most of the studies employing fNIRS in PD patients, focused on the prefrontal cortex activation during walking or dual tasks^12,13^, while, to the best of our knowledge, this is the first study exploring the mode of motor network activation before and during others’ movement observation. In patients with a different type of disease, i.e. fibromyalgia, we found a basal ipo-metabolism of the motor cortex, probably due to a persistent inhibition from chronic pain^14^. In PD patients, at least in the early phase of the disease, the motor network displayed a normal metabolism. However, in accordance with EEG data, alpha mu desynchronization prevailed in PD patients on the left fronto-central regions, in the resting state before the task requiring a behavioural motor reaction. In PD patients, the preparation for active movement could request additional resources with respect to controls, so we could not exclude that compensatory phenomena of cortical activation may support motor reaction.

### Time-to-contact detection session

 The computation of the total values of oxyhemoglobin levels on the left and right hemispheres during the tasks showed that in the task requesting an active reaction, the vision of the hand grasping the flat object determined a stronger activation of the left and right motor networks in both groups, in comparison with the sharp tip object. This is in line with the results originally obtained by Craighero et al.^7^ using TMS to demonstrate motor cortex facilitation during the vision of the suitable movement. These results could demonstrate substantial integrity of motor resonance circuits in PD patients, at least in the early stage, in line with the consequent facilitation of motor reaction. Single-channel analysis indicated a metabolic activation of the right hemisphere in control subjects, and of both hemispheres in PD patients. There was no significant trend of increased activation of the left hemisphere during the congruent movement observation in PD patients. This phenomenon of increased activation of motor circuits during others’ correct movement observation, and its possible role in the global strategies of motor programming in PD, deserves confirmation in larger series and in a more advanced stage of the disease.

The time–frequency analysis showed a desynchronization of alpha rhythm in the 1 s time preceding and following the movement observation, which was similar for the 2 objects and for the 2 groups. This is in line with previous studies, showing that changes in EEG mu activity provide a valid means for the study of human neural mirroring^15^.

Similarly to fNIRS results, in both PD and control groups, we observed a prevalent alpha mu desynchronization in the time preceding the vision of the flat object grasping. The desynchronization was represented on the parieto-occipital and central electrodes in controls and left prefrontal and posterior central electrodes in patients. No significant differences were detectable between groups with regard to the spatial distribution of the desynchronization induced by the congruent movement. Contamination with occipital alpha suppression is possible during a visual task^16^, and the lack of topographic specificity of mu desynchronization may be a result of more general attention processes^15^. The contribution of neuroimaging methods, such as fMRI^17^ and fNIRS^18^ could further clarify the role of cortical regions involved in mirroring phenomena, as in the present study, in which an increase of oxyhemoglobin levels was detected in the motor network. The lack of statistical differences in alpha mu desynchronization modality between patients and controls is a confirmation of the substantial integrity of motor resonance mechanisms in early PD.

### Observation-only session

 In this task, we observed that the changes of oxyhemoglobin during the video observation were not relevant when considering the total values of left and right hemisphere channels. Topographic analysis of single channels revealed that in controls the congruent movement had an effect on the activation of left and right cortical regions, while this phenomenon seemed less evident in PD patients. However, we did not observe statistical differences between groups. The possibility that in PD patients motor resonance mechanisms could be prodromal to active reaction, in order to expedite and facilitate the performance of the movement, needs confirmation in larger groups. The alpha mu showed desynchronization in the second preceding and following the object grasping, in a more evident way in controls for the ungraspable object. This type of EEG phenomenon could be explained with a sort of mirroring activation due to other potential motor failures and was strictly time-related to the vision of the hand grasping. The alpha mu desynchronization is associated with execution more than observation^15^, and this could explain the contradictory results obtained with the 2 brain functional analysis methods. The phenomenon we observed in controls with the fNIRS method, consistent with a cortical activation induced by the more suitable movement, was computed in the global time of the task and not evident in the time–frequency EEG analysis which, on the other hand, displayed a time related cortical reaction to uncorrected movement, which was not detectable with the fNIRS method. In any case, parkinsonian patients seemed less reactive at the cortical level during the simple observation of movement, supporting the hypothesis that mirror phenomena could have a function of motor facilitation in patients with initial dysfunction of movement programming.

### General remarks

 As already reported in recent works, action observation therapy (AOT) has shown evidence of efficacy as a rehabilitation strategy in PD patients^19,20^. Such an approach for therapy, in fact, was revealed to be effective in both single-session experiments and long-term therapeutic programs^21,22^; in addition, a recent review work evidenced how this kind of approach was easier to apply respect to others, such as those based on Motor Imagery^23^. However, mirroring circuits activation seems weaker in PD patients during the observation of others’ gait, as compared to controls^3^.

Here we provide the first evidence of active motor resonance mechanisms in early PD, with active response facilitation obtained with the observation of movement with explicit semantic clues. Such phenomena could compensate for a possible initial failure in motor programming, as shown by the good performance PD patients demonstrated in motor reaction after the more suitable movement observation.

Based on the present results, we could suppose that modifying the content of action observation, in order to stimulate motor resonance with the use of congruent movement, could improve the efficacy of such rehabilitation strategies. Indeed, a recent work made evident the modulation induced by motor resonance in healthy subjects, linking such excitability to the efficacy of the AOT itself^24^.

In a recent study conducted with the same paradigm^11^ in chronic pain patients and controls, we found that in the latter group the vision of graspable and ungraspable object did not produce relevant changes in cortical activation during the observation and time-to-contact detection sessions. In that study, the control group included younger people, so we can assume that the cortical activation induced by the vision of other’s correct movement is a phenomenon facilitating active reaction in people with initial dysfunction of motor circuits, as in normal aging and the initial phase of extrapyramidal disorders.

## Methods

### Participants

The experimental study included 21 patients affected by early PD (age = 62.52 ± 9.6) and 22 healthy subjects (age = 59.06 ± 9.7) as control group. PD patients, whose clinical and demographic characteristics are reported in Table 1, were enrolled during routine clinical practice, at the Neurophysiopathology Unit of Bari Polyclinic General Hospital. Inclusion criteria were: diagnosis of idiopathic Parkinson's disease, Hoehn-Yahr stage < II, age between 40–80 years, MMSE > 24, absence of significant visual deficits. All patients were stable without motor/non-motor fluctuations and dyskinesias.

**Table 1:** Clinical and demographic characteristics of Parkinson’s disease patients.

Parameter	Value
Sex/Mean Age	13 M/59 y—8 F/63 y
Years from diagnosis	4.8 y (mean), 1–12 years (range)
MMSE	27.8 (mean), 25–30 (range)
H&Y stage (n° pts)	I (10)–I.5 (6)—II (5)
UPDRS III	12/108 (mean), 4–24/108 (range)
Phenotypes (n° pts)	Akinetic-rigid (9)—Tremor Dominant (12)
First Affected side (n° pts)	Left (6)—Right (10)—Bilateral (5)
Mean LDopa dose/day	300 mg/die, range 150–700 mg/die
Other PD therapies (n° pts)	IMAO-B: Rasagiline, Safinamide, Selegiline (14) DA: Rotigotine, Pramipexole, Ropinirole (7)
Patients taking LDopa only	4 Pts
Patients taking LDopa + IMAO-B/Dopamine Agonists	9 Pts LDopa + IMAO-B 2 Pts LDopa + DA 4 Pts LDopa + DA + IMAO-B
Patients taking IMAO-B or Dopamine Agonists only	1 Pt Rasagiline in monotherapy 1 Pt DA + IMAO-B without LDopa

All participants were consistent right-handers according to the Edinburgh Handedness Inventory. All participants provided written fully informed consent before entering the experimental study. The study was conducted in compliance with the Declaration of Helsinki and approved by the Ethics Committee of the Bari Polyclinic General Hospital. For each group, subjects with less than eight years of schooling, with major psychiatric diseases, diseases of the central or peripheral nervous system, diabetes, thyroid diseases, severe chronic kidney disease, autoimmune and connective system diseases, were considered not eligible for the study, together with cases using substances or drugs with CNS effects, excluding those specifically prescribed for PD (L-dopa, dopa agonists).

### Stimuli

We adopted the same stimuli of the study by Craighero et al.^7^. They consisted of two videos of the same duration (2640 ms), showing the same agent sitting at a desk executing a movement for reaching and grasping an object. The agent was recorded from a third-person perspective. In the “flat object video” the object consisted of a parallelepiped (width: 7 cm; height: 3 cm; length: 3 cm) placed with its longer axis facing the agent. Without lifting the object, the agent reached out and naturally grabbed the parallelepiped with her fingers parallel to her frontal plane. Using a software for video editing, the parallelepiped was changed to a polyhedron (with the same dimension of the parallelepiped, i.e., 7 cm × 3 cm × 3 cm) in the “sharp-tip object video”. In this way, the kinematics of the movement remained the same and the agent's fingers touched the object precisely at the sharp tips. The instant at which the experimenter’s index finger touched the object was the same for both videos (1880 ms, Frame 47). The two videos were further modified to produce two catch-trials videos stopped before the agent’s hand touched the objects (1520 ms after the beginning of the video, Frame 38). To achieve the same time duration as the experimental-trial videos (2640 ms), the last frame was repeatedly shown. Catch-trial videos were provided merely to make participants constantly pay attention to video content all the time; they were never taken into account while analyzing the results.

### Experimental procedure

A multimodal fNIRS-EEG co-registration system was used to conduct the study, as detailed in the following paragraphs. The participant was seated on a chair in a quiet room, in front of a desk on which there was a display (60 cm from the person) and a keyboard. Before the experiment began, each participant was asked to grab both objects shown in the video with the same grip used by the agent. This request was intended to demonstrate to the participants that the heavy weight of the object (240 g), and the presence of sharp tips right at the point of contact with the fingers made it impossible to grasp the sharp-tip object with the grip shown in the video. On the contrary, although the weight was the same, that type of grip was suitable for grasping the flat object. Participants were submitted to two experimental sessions: time-to-contact detection session, and observation-only session. Each experimental session consisted of 42 randomly presented trials: 30 experimental trials (15 flat object videos, and 15 sharp-tip object videos) and 12 catch trials (6 flat object catch videos and 6 sharp-tip object catch videos).

At the beginning of each experimental session, the participant stared at a fixed cross on the black screen for 120 s, to record 20 s baseline and 100 s resting state. They were informed about the type of session which was starting at the beginning of the resting-state recording, and warned again 5 s before the start of the videos. In fact, we intended to use the resting-state to detect, for both fNIRS and EEG, the modality of preparation to action observation. A black screen was shown between videos for 15 s. At the end of each experimental session, each participant was allowed 5 min of rest.

#### Time-to-contact detection session

The participant’s left arm was relaxed on the desk. They were told to watch the videos and use their right index finger to tap the space bar on the keyboard when the agent touched the target object (experimental trials); however, they were not allowed to tap the space bar when the agent's hand stopped before touching the target object (catch trials). The participant’s response to catch trials was counted and treated as an error. The subject was excluded from the analysis if the number of errors was 6 or higher. A small percentage of incorrect responses made sure that the task was executed considering the time of the touch and no further clues.

#### Observation-only session

 Both of the participant’s arms were relaxed on the desk. Participants were instructed to watch the videos carefully. Six times, at random, the following question appeared on the screen: “What object did you just see?”. Participants' responses were noted and verified by a researcher. If the participant made a number of errors equal to or greater than 3, they were excluded from the study sample. The error limit ensured that only participants with high levels of attention to the videos were considered.

### fNIRS system

**Figure 8:**
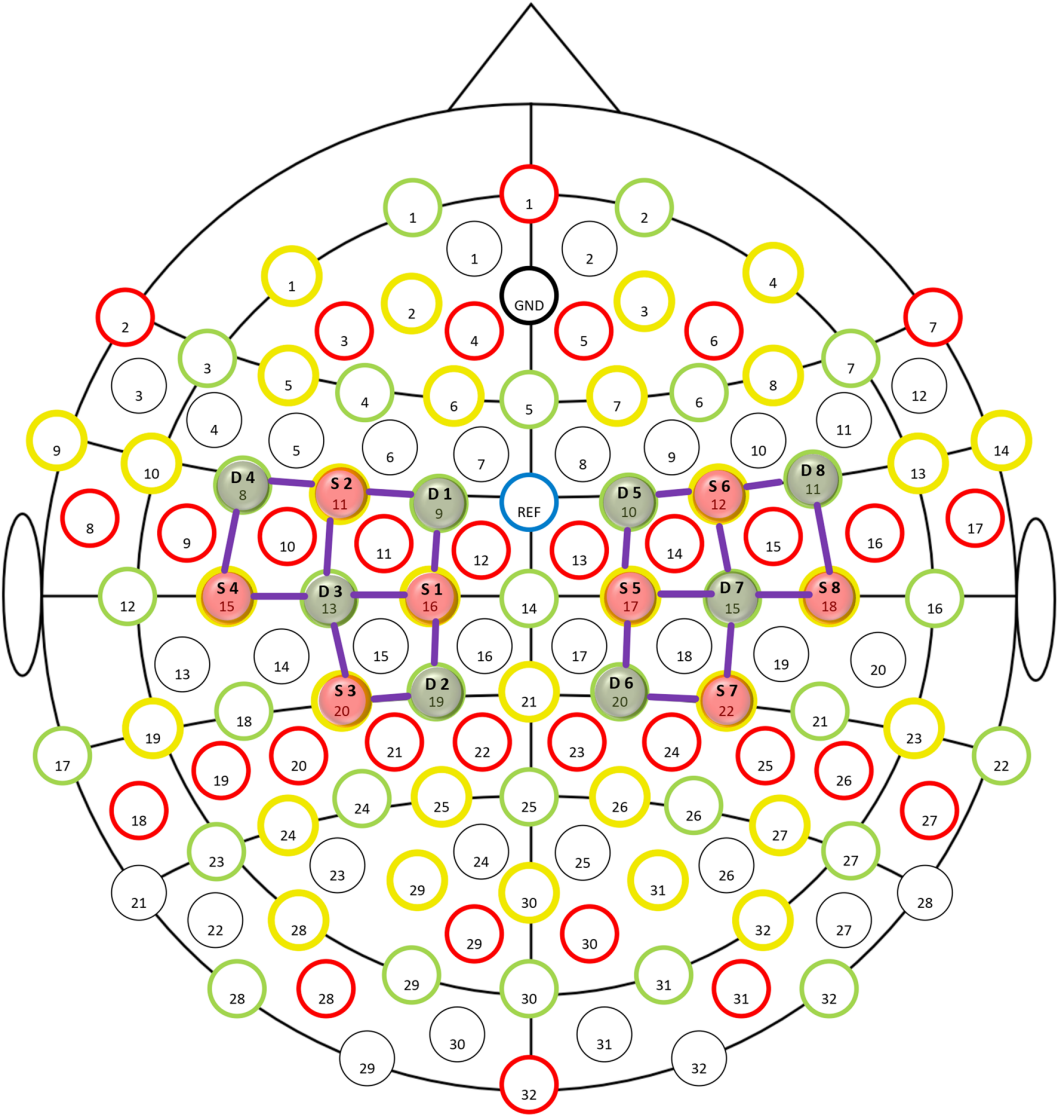
fNIRS channels design, 8 × 8, for motor cortex. S, source; D, detector.

We used a cap adapted to co-registration consisting of 20 fNIRS channels and 62 active Ag–AgCl surface electrodes (Fig. 8).

For this study, a continuous wave NIRS system (NIRSport 8 × 8, Nirx Medical Technologies LLC, Berlin, Germany) that captures brain oxygenation measurements was adopted. The fNIRS device is considered a promising tool for studying action observation mechanisms^25^. The device is a multi-channel system, easy to wear, and contains LED sources and photosensitive detectors (sensitivity: > 1 pW, dynamic range: > 50 dB). The data was recorded using NIRStar 14.2 software (Version 14, Revision 2, Release Build, 2016-04-15 NIRx Medizintechnik GmbH, Berlin, Germany; www.nirx.net). The experimental design involved the use of eight sources and eight detectors (commonly known as optodes). The NIR light source used in the system emits two wavelengths of 760 and 850 nm. The light absorption by brain tissues is wavelength-dependent and permits to estimate of different values for the oxyhemoglobin (ΔHbO2) and deoxyhemoglobin (ΔHb) respective concentrations during experimental sessions. A wavelength-dependent differential path length factor (DPF) is included in the modified Beer-Lambert law (MBLL) to simply determine the relative variations of the concentration of ΔHbO2 and ΔHb.

The optodes were positioned on selected portions of the scalp with the intent of evaluating the activity of a set of cerebral cortical regions relevant to the designed experiment; specifically, they were positioned over the primary and supplementary motor cortex (as shown in Fig. 8). As the previous evidence suggests, the ideal distance between sources and detectors was 30 mm for acquiring a good optical signal. The oxyhemoglobin and deoxyhemoglobin (ΔHbO2 and ΔHb) data were collected with a sampling rate of 7.81 Hz. However, in this study, we evaluated the ΔHbO2 levels only (see paragraph below). A sensor calibration procedure was carried out before every measurement. This digital practice allows ascertaining the adequate signal amplification for each optodes combination.

#### fNIRS signal processing

fNIRS signal processing was made using nirsLAB MATLAB-based software (nirsLAB, version 2017.06, NIRx Medical Technologies, Glen Head, NY, USA). Specifically, the researchers performed a raw signal cleaning process with the following functions: removing discontinuities from the signal, removing peak motion artifacts, baseline correction, and hemoglobin molar extinction coefficients.

The analysis was carried out on 8-s-long epochs, where each epoch began at the start of each video. For digital filtering, a 6th-order Butterworth Low Pass Filter with cut-off frequencies of 0.06–0.2 Hz was applied to raw data to eliminate the respiratory and cardiac frequencies from the signal in each recording channel. The oxyhemoglobin levels changes were computed, as a reliable measure of cortical metabolic status^26^. To calculate the molar extinction coefficients of hemoglobin, the researchers adopted the spectrum published by W.B. Gratzer (Med. Res. Council Labs, Holly Hill, London) and N. Kollias (Wellman Laboratories, Harvard Medical School, Boston, MA, USA). Then, the optical intensity data were converted into ∆HbO2 (in mmol/liter) concentration by the modified Beer-Lambert law.

Before the application of the modified Beer-Lambert law, for each participant, a baseline corresponding to the first 20 s of each recording was subtracted. Block-average fNIRS responses were then calculated, after subtracting baseline averages of each block, i.e., 5 s before stimulus onset.

### EEG

The EEG signal was acquired using the Micromed Brain Quick equipment at a sampling rate of 256 Hz using 61 electrodes positioned according to the 10–10 international system. To acquire also the electrooculogram (EOG), two electrodes were placed on the right and left eyebrows, respectively. The reference electrode was positioned on the nasion, and the ground electrode on Fpz. A 0.1–70 Hz band-pass filter with a 50 Hz digital filter was applied during the EEG recording. The EEG was recorded during the entire experimental procedure.

#### EEG signal processing

The EEG data preprocessing was performed by adopting EEGLAB 14.1.1, a MATLAB-based software^27^. The researchers used a semi-automatic method based on visual detection and channel statistics to locate and remove the faulty recording channels. All channels with distributions far from the Gaussian one were excluded from the analyses. Ocular artifacts recorded by the EOG channels were removed by means of the ICA algorithm included in the EEGLAB tool. Next, all the EEG files were processed using Letswave 7 tool (https://letswave.cn). EEG has been re-referenced to 0 value and pre-filtered with a band-pass filter in the range[1–24] Hz.

To evaluate the not phase-locked synchronization/desynchronization of alpha mu and beta mu, the researchers used a time–frequency analysis based on Continuous Wavelet Transform (CWT)^28^, with a baseline correction computed on the 20 s preceding the resting-state. The absolute power of the alpha (7–12 Hz) and beta (13–30 Hz) bands were considered for the single experimental conditions.

In order to detect the preparation to action observation, epochs lasting 5 s that preceded both the start of the observation-only session and the time-to-contact detection session were considered. To evaluate the alpha mu changes, with respect to the baseline, related to the vision of the flat and sharp tip objects, we computed the CWT in a time window from 2 s preceding the object grasping to 1 s following it, so the EEG was recorded simultaneously to the movement of the arm in the video.

### Statistical analysis

#### Behavioural data

The considered dependent variable was the time lag between the instant at which the agent’s index finger touched the object (Instant of Touch), i.e., 1880 ms from the beginning of each video, and the participant’s key pressing (Response). For each participant, for each trial, we calculated the time lag as Instant of Touch-Response. The time lag was submitted to a two-way ANOVA with Object (flat object vs sharp-tip object) as the within-subject variable and Group (PD patients vs Controls) as the between-subjects variable.

#### fNIRS

Before starting each recording, the participant's age-dependent Differential Path-length Factor (DPF) was entered into the signal acquisition software. To study changes in cerebral hemodynamic activity during tasks, the mean of the change in ∆HbO2 was calculated for each experimental condition. According to our previous study^11^, the analysis window chosen for each event was 8 s. The GLM method was applied to investigate the activation in brain regions of interest. Therefore, for each experimental session, the hemodynamic response function (HRF) was adopted to model the fNIRS signal. SPM-1 within-subject analysis allowed for estimating the activation (beta values) in each fNIRS channel with respect to the baseline. A two-way ANOVA was also applied to evaluate the oxyhemoglobin levels on the averaged values of left and right fNIRS channels, using groups and sessions as factors in the resting state, and groups and objects as factors in the observation-only and time-to-contact sessions.

For behavioral data and Oxyhemoglobin levels on single channels, we used the IBM Statistics Package for the Social Sciences (SPSS), Version 28.0 (IBM Corp., Armonk, NY, USA). For all analyses, the significance level was set at 0.05.

#### EEG

For topographical analysis and generation of Statistical Probability Maps, we used Matlab Letswave 7 tool, applying the Student’s t-test for paired data to compare the absolute power of alpha power in single groups. The two-way ANOVA with conditions and groups as factors was also applied, to establish differences of alpha mu behaviour in resting-state preceding observation and time-to-contact detection sessions and during these sessions between flat vs sharp tip object grasping conditions. To overcome the multiple comparisons problem, we performed the statistical analyses performing a nonparametric cluster-based permutation approach^29^. The calculation of the cluster-based statistics consists in grouping together neighboring t-values obtained for (frequency, time)-samples into clusters and summing the statistical values within each cluster. For inclusion in a cluster, only statistical values higher than the cluster-forming threshold, which was set to 0.05, are considered. Then, the significance probability is calculated with a Monte Carlo approximation based on the number of permutations. As a rule of thumb proposed in previous works^30,31^, this number should be no less than 1000. Thus, to perform feasible computations, we set it to 2000. For representation purposes, the Statistical Probability Maps show the significant results obtained after permutations in the range 0.001–0.01.

## Study limitations

The study included a small number of patients, and results deserve further confirmation in different stages of the disease and possible correlation with clinical features. The effect of L-dopa treatment could also be tested. The NIRS analysis was limited to the motor cortical areas, but the EEG revealed possible activation of other regions outside the sensory-motor network, whose real significance in mirroring phenomena in PD patients remained unclear. Although this result could reveal to be an overestimation of the real effects, due to the assumptions done to overcome the multiple comparisons problem, the cluster-based approach could guarantee a sufficient level of reliability of the analyses carried out.

Lastly, we considered the oxyhemoglobin values, which have demonstrated a good performance in fNIRS analysis, but there is no general accord with the best method for the evaluation of cortical metabolic changes.

## Conclusion and future perspective

This study demonstrates the substantial preservation of motor resonance mechanisms in early PD patients. The possibility that the action observation finalized to a consequent movement can activate cortical networks in patients with no advanced motor limitations, allows us to envisage the future design of early rehabilitation interventions taking into account specific observation paradigms, and preceding motor production.

## Data Availability

The datasets used and/or analysed during the current study are available from the corresponding author on reasonable request.
